# Social connectedness and mental health before and during the COVID-19 pandemic in a community sample in Korea

**DOI:** 10.1371/journal.pone.0292219

**Published:** 2023-10-19

**Authors:** Sojung Lee, Hyejoo Moon, Jisu Ko, Banu Cankaya, Eric Caine, Sungeun You

**Affiliations:** 1 Department of Psychology, Chungbuk National University, Cheongju, South Korea; 2 Department of Psychology, MEF University, Istanbul, Turkey; 3 Department of Psychiatry, University of Rochester Medical Center, Rochester, New York, United States of America; The University of Sydney, AUSTRALIA

## Abstract

This study compared social connectedness patterns and examined the relationships between objective or subjective social connectedness and mental health before and during the COVID-19 pandemic among community dwelling adults in South Korea. An identical online survey was administered at two time points, in 2019 prior to the onset and again in 2021. Objective (network diversity and network size) and subjective (thwarted belongingness and perceived burdensomeness) social connectedness were measured along with positive and negative indices of mental health (depression, suicidal behavior, happiness, and life satisfaction). The results indicated that among social connectedness indices perceived burdensomeness were significantly higher during the COVID-19 pandemic compared to the prior period, while network size was smaller. Subjective social connectedness was associated with all aspects of mental health consequences, either positive or negative. Among objective social connectedness, only network diversity was significantly associated with increased happiness and life satisfaction, and objective social connectedness was not associated with depression and suicidal behavior. These associations did not differ across the two time periods. The findings, both before and during the pandemic, indicated that network diversity is an important factor for positive indices of mental health and that efforts to increase subjective social connectedness are needed to decrease the risk of depression and suicidal behavior.

## Introduction

Social connectedness is widely recognized as a potent determinant of mental health [1–3]. According to Baumeister and Leary [4], human beings have a basic need for social connection and belongingness. People have a desire to establish and maintain appropriate interpersonal relationships, as meeting this need evokes positive emotions and reduces stress [4]. If this need is unmet, negative mental health consequences follow [5–7]. Deterioration of social connectedness can worsen the symptoms of depression [7, 8] and lead to suicidal thoughts, increasing the risk of suicide [9–12]. Also, it decreases one’s life satisfaction or happiness [5, 6, 9, 10]. Since the World Health Organization announced the COVID-19 pandemic in March 2020, numerous countries adopted prevention measures of social distancing and quarantining. While social distancing and quarantining slowed the spread of the virus, they markedly reduced face-to-face human interactions. Such forced restriction during the pandemic has reduced social connectedness and possibly leads to negative consequences in terms of mental health [11].

Social connectedness characterizes the degree to which one connects to other people [12]. This term is commonly used in psychiatry, psychology, and sociology, but no standard definition or agreed-upon concept exists [13]. Berkman’s seminal article on social integration and health suggest a multi-level structure of social relationships, in which social connections are conceptualized from a macro-level contextual factors to micro-level psychobiological processes [14]. Berkman’s model depicts how we conceptualize social connectedness from an objective, structural level to subjective, psychological level. Consistently, Cornwell and Waite [15] suggested that social connectedness can be conceptualized by dividing it into objective and subjective domains. Objective social connectedness describes the structural aspects of social connection, such as social network size or diversity [16]. Network size refers to the number of people in one’s social network and network diversity refers to the number of types of social network [16]. Subjective social connectedness reflects subjective perceptions, including loneliness or thwarted belongingness [15]. Research has shown that these two domains of social connectedness exhibit a low degree of correlation [17, 18], suggesting that they are, though related, independent constructs [15]; thus, the intervention methods for the two are also different [19].

Considering objective and subjective social connectedness as distinct concepts raises the question whether they have differential impacts on a person’s mental health, especially for negative and positive aspects of mental health. It has been reported that negatively perceived subjective social connectedness correlates more strongly with the presence of psychopathology compared to measures of objective social connectedness [15, 18, 20]. Objective measures, such as isolation or diverse social activities were more related to psychological well-being and hedonic emotions [21, 22].

The COVID-19 pandemic has served to present a ‘natural experiment’ that has allowed further exploration of these issues. Multiple reports have indicated that feelings of isolation and loneliness have increased relative to the pre-COVID period and that the likelihood of developing depression, panic symptoms, and emotional disturbances has increased [11, 23, 24]. Also, a study conducted during the pandemic showed that thwarted belongingness predicted psychological distress [25]. Loneliness and a reduced social network were significantly related to depression, anxiety, and stress [26]. Furthermore, staying at home due to coronavirus infection was associated with increased suicidal thoughts or behaviors [27]. These findings suggest perceived social disconnection during the pandemic was associated with negative aspects of mental health such as depression and suicidal thoughts. Yet, the relationship between social connectedness and mental health since the COVID-19 pandemic remains relatively unexplored. In addition, the measurement of objective aspects of social connectedness often was neglected in previous studies. However, it needs to be appropriately measured to reflect the challenge posed to linking with other people during lockdowns and distancing due to the pandemic.

Studies comparing social connectedness patterns before and during the pandemic are limited. Mostly, previous studies have described the relationship between social connectedness and mental health during the pandemic [26–29] or compared differences in social connectedness and mental health before and during the COVID-19 pandemic using retrospective data collected during the pandemic [30]. Additionally, studies regarding social connectedness and mental health before and during the pandemic have either measured only one aspect of subjective social connectedness [24, 31] or have not differentiated objective and subjective aspects of social connectedness [32].

The pandemic has led to health-related policy decisions of forced isolation and the impact of such preventive measures are largely unknown. This study aimed to investigate the relationship between social connectedness and mental health using two datasets collected separately at two time points, one in 2019 before the COVID-19 pandemic and the other in its midst during 2021. The goals of this study were as follows. First, we sought to examine whether the levels of the objective and subjective indices of social connectedness and mental health would differ between the two time periods; that is, before and after the height of the COVID-19 pandemic in South Korea. We hypothesized that both objective and subjective social connectedness would be lower after the onset of the pandemic; we hypothesized that depression and suicidal behavior would be higher while happiness and life satisfaction would be lower after the pandemic than the prior period. Additionally, we examined whether contact frequency in diverse social network types would differ between the two, to explore differences in social network patterns before and after the pandemic onset. Second, we aimed to examine whether objective and subjective social connectedness would function differentially on negative outcomes (i.e., depression and suicidal behavior) and positive outcomes (i.e., happiness and life satisfaction) of mental health, and the associations between social connectedness and mental health would differ across the pre- and during the COVID-19 pandemic.

## Materials and methods

### Participants and procedures

Eligible participants included community dwelling adults in South Korea, ages 19 to 50 years. They were recruited via social networking platforms or online communities. A link to the online survey was provided to those who consented to participate. An identical survey was administered using the same methodology in 2019 and 2021. A total of 358 adults participated in the 2019 survey, and among them, 296 responses were retained after excluding careless responses. We identified careless responses if the participants provided the same responses to all or most questions, response time was unreasonably short, and inconsistent demographic information was detected within the survey (e.g., gender, marital status). There were 326 participants in the 2021 survey, and 291 responses were retained. Female participants significantly outnumbered males in both samples (68.2% in 2019 and 73.2% in 2021). The mean age was 25.7 years (SD = 5.40) and 26.5 years (SD = 7.6) in the samples in 2019 and 2021 survey participants, respectively. Most had low monthly incomes of less than 1 million KRW, approximately 1300 USD (55.1% in 2019 and 55.7% in 2021), identified them as students (49.7% in 2019 and 50.5% in 2021), had permanent employment (27.7% in 2019 and 28.2% in 2021), possessed university degrees (67.9% in 2019 and 66% in 2021), and reported living alone (68.9% in 2019 and 77.3% in 2021). No significant differences were found in demographic variables between the two samples except for living status; more participants in 2021 reported living alone compared to those in 2019 (Table 1).

**Table 1:** Demographic characteristics of the sample before and during the COVID-19 pandemic.

	2019 *N* = 296		2021 *N* = 291		Statistics	*p*
	*n*	%	*n*	%		
Gender					*x*^2^ = 1.74	.187
Men	94	31.8	78	26.8		
Women	202	68.2	213	73.2		
Age (*M*, *SD*)	25.7	5.40	26.5	7.63	*t* = 1.48	.140
Income (KRW ^a^)					*x*^2^ = 7.37	.194
≤1,000,000	163	55.1	162	55.7		
1,010,000~1,500,000	16	5.4	19	6.5		
1,510,000~2,500,000	67	22.6	45	15.5		
2,510,000~4,000,000	37	12.5	47	16.2		
4,010,000~6,500,000	9	3.0	15	5.2		
> 6,5100,000	4	1.4	3	1.0		
Job status					*x*^2^ = 3.33	.853
Full-time employee ^b^	82	27.7	82	28.2		
Part-time employee	21	7.1	19	6.5		
Self-employer	7	2.4	6	2.1		
Unemployed	14	4.7	14	4.8		
Retired	4	1.4	1	0.3		
Housewife	2	0.7	5	1.7		
Students	147	49.7	147	50.5		
Others	19	6.4	17	5.8		
Education					*x*^2^ = 0.24	.620
Less than high school	95	32.1	99	34.0		
College degree or higher	201	67.9	192	66.0		
Living status					*x*^2^ = 5.26	**.022**
Living alone	204	68.9	225	77.3		
Living with others	92	31.1	66	22.7		

Note

^a^ 1 dollar = 1300 KRW

^b^ Full-time employee with insurance coverage

All study protocols were approved by the institutional review board at Chungbuk National University (CBNU-201812-SB-0211 for 2019 data collection; CBNU-202102-HR-0002 for 2021 data collection). All participants provided written informed consent online prior to their participation in the study.

### Measures

#### Measures of social connectedness

*Objective social connectedness*. The Social Network Index (SNI) [16] was used to measure objective social connectedness. The SNI measures social networking in terms of whom one has met or connected with during the past two weeks. The SNI assesses objective social connectedness in twelve types of social networks: spouse, parents, parents-in-law, children, other close family members, friends, religious group members, colleagues, schoolmates, neighbors, fellow volunteers, and others from the social club or recreational group. With the exception of the spouse type, how many people in each network type were contacted over the past two weeks were assessed. For the spouse type, the presence or absence was assessed. One doctoral level psychologist and two graduate students first translated the scale into Korean and a professional translator was hired to back-translate the items into English. After reviewing the translation and back-translation, the final items were confirmed. This study used the scoring criteria suggested by Cohen et al. [16] and Bickart et al. [33] in calculating network diversity and network size. Network diversity refers to the number of types of social networks that one connects with at least every other week, and network size refers to the number of people in the social network with whom one connects at least every other week. The social network size more than 100 was recoded as 100 to adjust extreme responses (*n* = 10). The reliability of the SNI, as evaluated using the Intraclass Correlation Coefficient (ICC), was found to be .70 [34].

*Subjective social connectedness*. The Interpersonal Needs Questionnaire (INQ-R) [35] was used to measure subjective perception in social connection. The INQ-R is a self-report scale to measure subjective social connectedness. Its sub-factors include thwarted belongingness and perceived burdensomeness. Thwarted belongingness measures psychological distress from having an unmet desire to connect with other people. Higher scores indicate a greater degree of thwarted belongingness. Perceived burdensomeness is the degree to which one feels like a burden on others, relating to the self-recognition of being helpless or incompetent in interpersonal relationships. The higher the degree, the higher the perceived burdensomeness. The measure consists of 15 items, and participant responses were recorded on a 5-point Likert scale ranging from 1, Strongly Disagree, to 5, Strongly Agree. This measure was selected because it includes perceived burdensomeness that has known as a strong risk factor for suicidal behavior [9], in addition to thwarted belonging, a broader concept of subjective social connectedness that are associated with mental health [25]. The internal consistency for the Korean version was 87–.88 [36], and we measured this value at .88–.92 in this study.

#### Measures of mental health

*Depressive symptoms*. The Center for Epidemiologic Studies Depression Scale (CES-D) was used to measure the symptoms of depression. The CES-D is a 20-item self-report scale developed by Radloff [37]. This study used the Korean version of the CES-D [38]. Responses experiencing the symptoms of depression were collected on a 5-point Likert scale, ranging from 0, Strongly Disagree, to 4, Strongly Agree. The internal consistency for this scale was at .91, and we found it to be .86 in this study [38].

*Suicidal behavior*. The Suicidal Behavior Questionnaire-Revised (SBQ-R) was used to measure suicidal behavior [39]. This study only used questions asking about suicidal behaviors (ideation, plan, and attempt) within the past year. The score ranges from 1 to 4, indicating 1 for no suicidal behavior, 2 for suicidal ideation, 3 for suicidal plan, 4 for suicidal attempt. The higher the score, the more serious the suicidal behavior. The internal consistency of the SBQ-R was reported as .88 [39].

*Happiness*. The happiness question used by the World Values Survey, “Overall, how happy are you?” was used to measure happiness [40]. Responses were given to the item on a 4-point Likert scale, ranging from 1, Very Unhappy, to 4, Very Happy. This item has been commonly employed in numerous happiness studies [41] and exhibited high concurrent validity [42] as well as predictive validity [43, 44].

*Life satisfaction*. Satisfaction with Life Scale (SWLS) was used to measure overall satisfaction with life [45]. This scale consists of five items on a 5-point Likert scale, ranging from 1, Strongly Disagree, to 5, Strongly Agree. The internal consistency of a Korean version of the life satisfaction scale was .84–.91 (Cronbach’s α) [46], and it was validated at .83 in this study.

### Statistical analysis

JAMOVI 1.6.23 was used for the statistical analysis. First, descriptive statistical analysis was performed on participants’ demographic information, depression, suicidal behavior, happiness, and life satisfaction. Next, an independent-samples *t*-test and a χ^2^ analysis were used to compare the difference between the 2019 and 2021 samples. Second, an independent- samples *t*-test was performed to investigate the differences before and during the COVID-19 pandemic in social connectedness and mental health indices. Additionally, an independent- samples *t*-test was performed to investigate the differences before and during the COVID-19 pandemic in social network types with whom one connected for two weeks. Third, hierarchical regression analysis was performed to examine the relationship between mental health and social connectedness (i.e., network diversity, network size, thwarted belongingness, and perceived burdensomeness). At the first stage (Model 1), the basic model, whose inputs were the control variables was verified. Based on the previous research [29, 47–49] demographic variables that are associated with mental health (i.e., age, gender, income level, education level, and living alone) were used as control variables. Next, during the second stage of the process, network diversity, network size, thwarted belongingness, perceived burdensomeness, and time were included in as inputs into the full model (Model 2). In the final model (Model 3), the interaction terms of four social connectedness indices and time were inserted to verify the hypothesis whether the associations between social connectedness indices and mental health differ across the pre- and during the COVID-19 pandemic. Four hierarchical regression analyses were performed to predict depression, suicidal behavior, happiness, and life satisfaction, respectively.

## Results

### Social connectedness and mental health before and during the COVID-19 pandemic

As presented in Table 2, among objective social connectedness, network size was significantly lower during the pandemic than the prior period (*t* = 3.29, *p* < .001) while no significant difference in network diversity was found between the two time periods. For subjective social connectedness, perceived burdensomeness was significantly higher during the pandemic than before (*t* = 2.00, *p* = .046) and thwarted belongingness had a nonsignificant trend to be higher during the pandemic than before (*t* = 1.90, *p* = .058).

**Table 2:** Social connectedness and mental health before and during the COVID-19 pandemic.

	2019		2022		*t*	*p*
	*M*	*SD*	*M*	*SD*		
Social Connectedness						
Network diversity ^a^	3.93	1.51	3.87	1.51	0.50	.615
Network size ^a^	18.59	19.68	14.00	13.53	3.29	**.001**
Perceived burdensomeness ^b^	10.05	4.03	10.81	5.14	2.00	**.046**
Thwarted belongingness ^b^	21.39	6.64	22.43	6.70	1.90	.058
Mental Health						
Depression ^c^	19.86	9.46	20.47	9.95	0.75	.801
Suicidal behavior ^d^	1.41	0.71	1.54	0.79	2.16	**.031**
Happiness ^e^	2.95	0.57	2.89	0.65	1.30	.193
Life satisfaction ^f^	14.72	3.57	14.90	3.97	0.60	.548

*Note*.

^a^ Social Network Index

^b^ Interpersonal Needs Questionnaire Revised (INQ-R) subfactors

^c^ Center for Epidemiologic Studies Depression Scale (CES-D)

^d^ Suicidal Behavior Questionnaire-Revised (SBQ-R)

^e^ World Values Survey

^f^ Satisfaction with Life Scale

Among mental health indices, only suicidal behavior was more prevalent during the pandemic (*t* = 2.16, *p* = .031); 28.7% (*n* = 85) of 2019 participants reported suicidal behavior while 37.4% (*n* = 109) of 2021 sample affirmed them (χ^2^ = 5.07, *p* = .024). No significant differences were detected between before and during the pandemic for depression, happiness, or life satisfaction (see Table 2).

Additionally, comparisons of contact frequency by social network type between 2019 and 2021 samples showed that the contact frequency with friends, schoolmates, members of one’s religious group and other group was significantly lower during the COVID-19 pandemic than before (Table 3).

**Table 3:** Contact frequency by social network type before and during the COVID-19 pandemic.

	2019		2021			*p*
	*M*	*SD*	*M*	*SD*	t	
Parents	1.68	0.57	1.63	0.58	-1.48	.258
Parents-in-law	0.16	0.51	0.13	0.44	1.13	.527
Children	0.11	0.55	0.17	0.54	-0.95	.338
Other close family member	0.83	1.89	0.88	1.52	0.63	.696
Friends	4.72	3.86	3.64	3.01	-0.39	**< .001**
Members of religious groups	1.23	5.11	0.52	1.88	3.80	**.026**
School mate	2.03	3.45	1.51	2.56	2.23	**.041**
Coworkers	0.84	1.75	0.68	1.30	2.05	.207
Close neighbors	0.23	0.80	0.28	0.95	1.26	.533
Fellow volunteers	0.24	1.09	0.09	0.68	-0.62	.050
Other group	7.03	18.30	4.44	12.5	2.00	**.046**

*Note*. Among 12 social network types, spouse was excluded from the analysis because it measures the presence or absence of spouse

### Objective and subjective social connectedness on mental health before and during the COVID-19 pandemic

As presented in Table 4, after controlling for demographic variables, thwarted belongingness and perceived burdensomeness, subjective indices of the social connectedness, were significantly associated with all aspects of mental health, either positive or negative (all *p*s < .01). Objective social connectedness indices, on the other hand, were not significantly associated with depression and suicidal behavior. Instead, among objective social connectedness indices, network diversity was associated with life satisfaction (B = .42, *t* = 4.28, *p* < .001) and happiness (B = .03, *t* = 2.01, *p* = .045). No interaction between social connectedness and time was significant, indicating the association of social connectedness and mental health did not differ across the two time periods.

**Table 4:** Hierarchical regression analysis of social connectedness on mental health before and during the COVID-19 pandemic.

	Model 1					Model 2					Model 3				
Variables	B	s.e.	95% C.I.	*t*	*p*	B	s.e	95% C.I.	*t*	*p*	B	s.e.	95% C.I.	*t*	*p*
DV1: Depression															
Gender	1.45	0.89	-0.30, 3.21	1.63	.104	1.43	0.73	0.00 2.85	1.96	.050	1.46	0.73	0.03, 2.90	2.01	.100
Age	-0.01	0.08	-0.16, 0.15	-0.11	.910	-0.02	0.07	-0.15, 0.11	-0.25	.802	-0.02	0.07	-0.15, 0.11	-0.28	.778
Income	-0.61	0.37	-1.33, 0.11	-1.66	.098	-0.07	0.30	-0.66, 0.52	-0.23	.819	-0.07	0.30	-0.66, 0.52	-0.23	.820
Education	-0.52	0.91	-2.32, 1.27	-0.57	.569	-0.58	0.75	-2.05, 0.89	-0.77	.440	-0.57	0.75	-2.04, 0.91	-0.75	.451
Living alone	0.87	0.90	-0.91, 2.64	0.96	.337	-0.32	0.74	-1.77, 1.14	-0.43	.670	-0.29	0.74	-1.75, 1.17	-0.39	.700
Network diversity						0.04	0.26	-0.47, 0.55	0.16	.874	-0.37	0.76	-1.86, 1.13	-0.48	.629
Network size						0.03	0.02	-0.02, 0.07	1.21	.226	-0.02	0.06	-0.15, 0.11	-0.31	.755
Perceived Burdensomeness						0.90	0.09	0.73, 1.08	10.35	**< .001**	0.75	0.30	0.16, 1.34	2.50	**.013**
Thwarted Belongingness						0.35	0.06	0.22, 0.47	5.59	**< .001**	0.26	0.20	-0.13, 0.64	1.31	.192
Time						-0.42	0.66	-1.71, 0.88	-0.63	.528	-4.31	3.11	-10.41, 1.80	-1.39	.167
Network diversity * time											0.27	0.48	-0.68, 1.21	0.56	.578
Network size * time											0.04	0.05	-0.05, 0.13	0.79	.430
Perceived Burdensomeness * time											0.09	0.18	-0.26, 0.44	0.51	.607
Thwarted Belongingness * time											0.06	0.12	-0.18, 0.31	0.50	.621
	*R*^2^ = 0.18, *F*(5, 581) = 2.07, *p* = .068					Δ*R*^2^ = 0.34, *F*(5, 576) = 63.06, *p* < .001					Δ*R*^2^ = 0.00, *F*(4, 572) = 0.60, *p* = .662				
DV2. Suicidal behavior															
Gender	0.21	0.07	0.08, 0.34	3.07	**.002**	0.20	0.06	0.08, 0.32	3.21	**.001**	0.20	0.06	0.07, 0.32	3.13	**.002**
Age	-0.01	0.01	-0.02, 0.00	-1.93	.054	-0.01	0.01	-0.02, 0.00	-2.16	**.031**	-0.01	0.01	-0.02, 0.00	-1.93	.055
Income	-0.04	0.03	-0.09, 0.02	-1.38	.167	-0.01	0.03	-0.06, 0.04	-0.35	.729	-0.01	0.03	-0.06, 0.04	-0.38	.706
Education	0.00	0.07	-0.14, 0.14	0.02	.984	0.01	0.06	-0.12, 0.13	0.11	.910	0.01	0.06	-0.12, 0.13	0.08	.934
Living alone	0.13	0.07	0.00, 0.27	1.94	.052	0.08	0.06	-0.05, 0.20	1.23	.219	0.08	0.06	-0.04, 0.21	1.27	.205
Network diversity						0.00	0.02	-0.05, 0.04	-0.09	.930	0.03	0.07	-0.10, 0.16	0.48	.633
Network size						0.00	0.00	0.00, 0.01	1.14	.254	-0.01	0.01	-0.02, 0.01	-0.92	.359
Perceived Burdensomeness						0.05	0.01	0.04, 0.07	7.01	**< .001**	0.07	0.03	0.02, 0.12	2.82	**.005**
Thwarted Belongingness						0.02	0.01	0.01, 0.03	2.93	**.004**	0.02	0.02	-0.02, 0.05	0.91	.364
Time						0.09	0.06	-0.02, 0.21	1.68	.094	0.23	0.27	-0.29, 0.75	0.86	.390
Network diversity * time											-0.02	0.04	-0.10, 0.06	-0.58	.561
Network size * time											0.01	0.00	0.00, 0.01	1.40	.162
Perceived Burdensomeness * time											-0.01	0.02	-0.04, 0.02	-0.80	.422
Thwarted Belongingness * time											0.00	0.01	-0.02, 0.02	0.00	.999
	*R*^2^ = 0.05, *F*(5, 581) = 6.26, *p* < .001					Δ*R*^2^ = 0.17, *F*(5, 576) = 26.33, *p* < .001					Δ*R*^2^ = 0.00, *F*(4, 572) = 0.75, *p* = .554				
DV3: happiness															
Gender	0.06	0.06	-0.05, 0.17	1.01	.312	0.04	0.05	-0.04, 0.13	0.99	.324	0.04	0.05	-0.05, 0.13	0.88	.380
Age	-0.01	0.00	-0.02, 0.00	-2.37	**.018**	-0.01	0.00	-0.02, 0.00	-3.01	**.003**	-0.01	0.00	-0.02, -0.01	-3.22	**.001**
Income	0.09	0.02	0.05, 0.14	4.06	**< .001**	0.06	0.02	0.02, 0.09	3.01	**.003**	0.06	0.02	0.02, 0.10	3.13	**.002**
Education	0.00	0.06	-0.11, 0.11	0.01	.989	0.05	0.05	-0.05, 0.14	0.97	.334	0.05	0.05	-0.04, 0.14	1.06	.291
Living alone	-0.14	0.06	-0.25, -0.03	-2.51	**.013**	-0.08	0.05	-0.17, 0.01	-1.80	.072	-0.09	0.05	-0.18, 0.00	-1.94	.053
Network diversity						0.03	0.02	0.00, 0.06	2.01	**.045**	-0.04	0.05	-0.13, 0.06	-0.75	.454
Network size						0.00	0.00	0.00, 0.00	1.35	.178	0.01	0.00	0.00, 0.01	1.69	.092
Perceived Burdensomeness						-0.03	0.01	-0.04, -0.02	-5.92	**< .001**	-0.01	0.02	-0.04, 0.03	-0.28	.782
Thwarted Belongingness						-0.04	0.00	-0.04, -0.03	-9.10	**< .001**	-0.04	0.01	-0.06, -0.01	-2.92	**.004**
Time						0.01	0.04	-0.08, 0.09	0.13	.900	0.06	0.19	-0.33, 0.44	0.29	.775
Network diversity * time											0.05	0.03	-0.01, 0.10	1.53	.128
Network size * time											0.00	0.00	-0.01, 0.00	-1.26	.210
Perceived Burdensomeness * time											-0.02	0.01	-0.04, 0.01	-1.46	.145
Thwarted Belongingness * time											0.00	0.01	-0.02, 0.02	-0.02	.987
	*R*^2^ = 0.19, *F*(5, 581) = 4.69, *p* < .001					Δ*R*^2^ = 0.34, *F*(5, 576) = 63.73, *p* < .001					Δ*R*^2^ = 0.00, *F*(4, 572) = 1.59, *p* = .175				
DV4: Life Satisfaction															
Gender	0.30	0.34	-0.38, 0.97	0.86	.388	0.12	0.27	-0.42, 0.65	0.42	.673	0.10	0.28	-0.44, 0.64	0.37	.711
Age	-0.07	0.03	-0.13, -0.01	-2.23	**.026**	-0.10	0.03	-0.15, -0.05	-3.81	< .001	-0.10	0.03	-0.15, -0.05	-3.74	< .001
Income	0.60	0.14	0.32, 0.87	4.22	**< .001**	0.35	0.11	0.12, 0.57	3.06	.002	0.34	0.11	0.12, 0.57	3.02	< .001
Education	-0.41	0.35	-1.10, 0.28	-1.17	.241	-0.02	0.28	-0.58, 0.53	-0.07	.941	0.00	0.28	-0.56, 0.55	-0.02	.988
Living alone	-0.77	0.35	-1.45, -0.09	-2.21	**.027**	-0.26	0.28	-0.81, 0.29	-0.93	.352	-0.26	0.28	-0.81, 0.29	-0.93	.353
Network diversity						0.42	0.10	0.23, 0.61	4.28	**< .001**	0.30	0.29	-0.26, 0.87	1.06	.290
Network size						-0.01	0.01	-0.03, 0.00	-1.63	.103	0.00	0.02	-0.05, 0.04	-0.18	.856
Perceived Burdensomeness						-0.15	0.03	-0.21, -0.08	-4.46	**< .001**	-0.20	0.11	-0.42, 0.03	-1.72	.086
Thwarted Belongingness						-0.26	0.02	-0.30, -0.21	-11.09	**< .001**	-0.20	0.07	-0.34, -0.05	-2.64	**.009**
Time						0.57	0.25	0.08, 1.06	2.29	**.023**	0.96	1.17	-1.35, 3.27	0.82	.414
Network diversity * time											0.08	0.18	-0.28, 0.44	0.44	.661
Network size * time											-0.01	0.02	-0.04, 0.03	-0.37	.715
Perceived Burdensomeness * time											0.03	0.07	-0.10, 0.16	0.47	.635
Thwarted Belongingness * time											-0.04	0.05	-0.13, 0.05	-0.91	.364
	*R*^2^ = 0.03, *F*(5, 581) = 4.72, *p* < .001					Δ*R*^2^ = 0.36, *F*(5, 576) = 70.27, *p* < .001					Δ*R*^2^ = 0.00, *F*(4, 572) = 0.28, *p* = .889				

*Note*. Demographics were coded as follows: Gender (men = 1, women = 2), Living alone (living with others = 0, living alone = 1), Education (Less than high school = 1, College degree or higher = 2); Time was coded as 1 for 2019 and 2 for 2021.

Additionally, demographic variables were not associated with depression or suicidal behavior, except that women were more likely to exhibit suicidal behavior (B = .21, *t* = 3.07, *p* = .002). On the other hand, younger age (B = -.01, *t* = -2.37, *p* = .018), higher income (B = .09, *t* = 4.06, *p* < .001), and living with others (B = -.14, *t* = -2.51, *p* = .013) were positively associated with happiness. Higher income (B = .60, *t* = 4.22, *p* < .001) and living with others (B = -0.77, *t* = -2.21, *p* = .027) were positively associated with life satisfaction. All data underlying the findings in this study are fully available without restriction (see S1 Data).

## Discussion

The present study examined the relationship between social connectedness and mental health before and during the COVID-19 pandemic. Social connectedness was measured by objective indices (i.e., network diversity and network size) and subjective perceptions (i.e., thwarted belongingness and perceived burdensomeness). Mental health indices include the negative indices (i.e., depression and suicidal behavior) and the positive ones (i.e., happiness and life satisfaction). Overall, the results of this study indicated that subjective social connectedness was associated with all aspects of mental health while objective social connectedness was only related to happiness and life satisfaction. There were no significant differences in these relationships between the pre-pandemic and pandemic samples.

First, we hypothesized that both objective and subjective social connectedness would be lower during the pandemic than the prior year due to the imposition of social distancing and quarantine public health protocols. This hypothesis was supported, in part. Our results indicated that network size was significantly smaller during the pandemic than the prior year whereas network diversity was not. Perceived burdensomeness was significantly higher during the pandemic than before, but thwarted belongingness was not. Overall, these results were consistent with previous studies conducted in western countries. Studies from the UK and the US reported an increased level of loneliness during the COVID-19 pandemic [50, 51]. A Canadian study also reported that perceived burdensomeness increased among college students during the COVID-19 pandemic [32]. A Swiss study reported that the number of social networks decreased during the COVID-19 pandemic in a student cohort [52].

South Korea did not enforce a strict lockdown but did restrict the number of people in essential and non-essential gatherings, and recommended working from home and restricting in-person school instruction and religious gatherings for more than two years. Thus, if one had diverse social groups, a person was more likely to have an opportunity for face-to-face social interactions than those who had not. This may have affected the result that social network size was smaller during the pandemic, but network diversity was not. Also, Korea has an advanced infrastructure for online communication and internet services. Thus, online chatting or text messaging have formed the primary way of staying connected, from even before the pandemic. During the pandemic, the ways that people stayed connected might have become slightly different, as video conferencing technology was quickly adapted; the amount of online networking has not decreased, especially among the young generation. Considering that most of the study sample were young adults who use various forms of online methods when connecting with others, this may have affected the result that thwarted belongingness was relatively intact during the pandemic. On the other hand, the economic crisis resulting from the COVID-19 pandemic may have contributed to higher levels of perceived burdensomeness during the pandemic. Perceived burdensomeness is a subjective feeling that one is a burden to others and is associated with economic difficulties, bankruptcy, or unemployment [53].

Second, we questioned whether objective and subjective social connectedness play different roles in positive and negative aspects of mental health and explored whether these relationships differed in our two samples. Consistent with previous studies [18, 24, 26], we found that measure of subjective, but not objective, social connectedness were related to depression and suicidal behavior. Our study found that perceived burdensomeness and suicidal behavior were significantly higher among our participants during the COVID-19 pandemic than those prior to the year. These findings are supportive, in part, with of our hypothesis, and consistent with the interpersonal theory of suicide [54]. According to this model, thwarted belongingness and perceived burdensomeness are significant contributors to suicidal thoughts and behaviors, with a meta-analytic study showing a stronger role for burdensomeness [9]. Similar to many parts of the world during the pandemic, numerous people in South Korea suffered economic loss because they lost jobs, had to close down their businesses, went bankrupt, or became unemployed [55]. Within the social context of economic loss or loss of their previous status during the pandemic, feeling that they are a burden on their family or people around them may have contributed to the development of depression and suicidal behavior. On the other hand, feelings of thwarted belongingness or perceived burdensomeness could be a consequence of depression or other deteriorated social circumstances [56, 57]. Also, they are prevalent in other mental health conditions such as anxiety disorder and personality disorders [58, 59]. Our study was unable to determine the causality of this relationship or account for other potential comorbid mental health conditions. Therefore, further investigations using a longitudinal cohort design would be beneficial to investigate the reciprocal relationship between social connectedness and mental health.

Our findings indicated that network diversity, as well as subjective social connectedness, played an important role in happiness and life satisfaction. This was consistent with previous studies, which have reported that subjective belief in belonging to the social network was associated with life satisfaction and positive emotions [60, 61], and forming connections with other people was related to the physiological mechanisms of emotions and stress responses, playing a critical role in subjective well-being [62, 63]. In this study, network diversity (i.e., having a variety of network resources) was associated with happiness and life satisfaction; by contrast, network size had no significant relation with happiness and life satisfaction. Aligned with our findings, Ali et al. [64] found that network diversity played a crucial role in the physical and mental health of the elderly; notably, the association between network size and the health of elderly individuals significantly reduced when accounting for network diversity. These results suggested that social benefits derived from having diverse social networks, rather than connecting with many people within limited types of social networks. Consistently, previous studies found that social engagements within a diverse network enabled individuals to access necessary and various resources [65], which promoted well-being and physical health [64, 66]. In fact, network diversity seems important because it can serve as an indicator of increased social engagement and the extent of available social resources. During the pandemic, South Korea implemented a social distancing policy that primarily aimed to reduce the size of gatherings. Thus, the greater the diversity of one’s social network, the more possibilities there were for engaging in social gatherings. This underscores the importance of network diversity, not merely its size, which becomes even more relevant, particularly during the pandemic due to the enforcement of social distancing policies.

Of note, although it was not a focus for this study, our results related to demographic variables were worth mentioning. Younger age, higher income, and living with others were significantly associated with increased levels of happiness and life satisfaction; however, they had no significant relationship with depression or suicidal behavior. This suggests that a positive economic and living status may improve positive aspects of mental health, while their impact on negative aspects is unclear. This finding, however, should be viewed cautiously, as the majority of this study sample were young women in their 20s or 30s and lived alone in the community in Korea. Thus, it awaits further investigation.

Additionally, we explored whether social contacts by network type would differ between the two time periods. We found that the contact frequency with family members, relatives, or colleagues did not significantly differ across the two time points, but connecting with close friends, schoolmates, and religious members was less frequent during the pandemic than before. These findings were consistent with the report in other countries that social connections with family were more or less similar between the pre- and during the pandemic whereas social connections with friends were significantly lower after the pandemic [67, 68]. A relationship with close friends or schoolmates is one of the most private connections, which involves personal and emotional proximity and functions as an essential resource for physical and mental health [69]. In addition, religious members are widely recognized social resources to help improve personal meaning of life and life satisfaction [70]. A nation-wide study conducted during the pandemic showed that connecting with friends reduced depression and anxiety [61, 71, 72]. Likewise, compared to students attending school, students taking online classes had higher levels of depressive symptoms and suicidal ideation [73]. Thus, our findings suggest that the prevention measure of social distancing differentially affected social connections by network type, with the potential risk of reducing ties with close friends, schoolmates, or religious members. The potential impact on mental health needs to be considered.

The findings of this study need to be considered within the cultural context. In East Asian countries, unlike in Western societies where prioritizing individual goals and internal desires is considered important for well-being, the values and demands of community, as well as individual goals and internal desires, are regarded as equally important for well-being [74]. This suggests that in Korean society, a sense of belonging and connection within the community might be more crucial for life satisfaction and happiness compared to Western societies. However, a cross-cultural comparison study found that loneliness, but not social network size, was associated with psychological distress during the pandemic across four countries including South Korea [75]. This is consistent with our findings and may suggest the importance of subjective social connectedness in negative outcomes of mental health during the pandemic across the cultural background. In addition, as Kawachi and Berkman [76] suggested, the effect of social connectedness on mental health may vary across gender, particularly in times of stress; lack of social support is more associated with women’s mental health during stressful periods. Due to the high rates of women participants in this study, we were unable to examine potential gender differences, and this warrants further investigation.

Finally, the results of this study did not find differences in the associations between social connectedness and mental health between the two time periods, before and during the COVID-19 pandemic. As this study was not a longitudinal investigation, we were unable to detect whether changes in social connectedness affected changes in mental health. We can only provide an overview of the general relationships between social connectedness and mental health at two time points. Thus, similar to the pre-pandemic period, during the pandemic, developing diverse social network resources is beneficial for happiness and life satisfaction while improving subjective social connectedness can buffer against deteriorated mental health, such as depression or suicidal behavior. From a clinical and public health perspective, it may be beneficial to allow for gathering and meetings, even small size ones, instead of implementing a complete lockdown during the pandemic. Utilizing online meetings could be helpful. In fact, several studies have shown that online interactions do not exhibit significant qualitative differences compared to offline interactions [77, 78]. Moreover, during the pandemic, there is a need for mental health service policies that address vulnerable groups in terms of social connectedness, such as individuals who have limited access or ability to use technology or live alone.

### Limitations

Several limitations need to be noted. First, we adopted a convenience sampling method using an online survey. This non-random sampling method along with the small sample size are limitations of this study. Thus, the results cannot be generalized to the broader population and instead interpreted within the study sample characteristics. The majority of our sample were in their 20s and 30s, students, and women. Thus, the results of this study primarily reflect the mental health conditions of the young population in South Korea, limiting their generalizability to other age groups or countries with different cultural backgrounds. In addition, around 11% to 17% of the participants did not complete the survey or provided potentially random responses and were thus excluded from the analysis. We were unable to identify the characteristics of those who were excluded from the study due to the careless responses and cannot rule out the potential impact of their exclusion on the results. Further research using a stratified sampling method would be helpful to confirm the findings. Second, this study was not a longitudinal cohort study and thus we were unable to explore within-person changes across the two time points. Although we collected data at two time points using the same method and the demographic characteristics of the two samples were not statistically different, the two samples are not the same individuals. There remains a possibility of the impact of some other factors that were not considered. Third, instead of implementing strict distancing measures in place, such as a lockdown or curfew, relatively relaxed distancing policies were practiced in Korea during the pandemic. It should be careful to generalize the findings to other countries that used different quarantining measures or different cultures, especially in social connections.

## Conclusion

Overall, the present study supported the importance of social connectedness on mental health. This study contributes to the field by exploring the roles of both objective and subjective social connectedness in relation to positive and negative outcomes of mental health. Particularly, this study highlights the importance of network diversity on happiness and life satisfaction and the importance of maintaining social connectedness to decrease the risk of depression and suicidal behavior. Therefore, when social networks are under threat, as in the COVID-19 pandemic, public policies should consider various approaches for fostering social connectedness and preserving network diversity while implementing social distancing measures.

## Supporting information

S1 Data Deidentified original dataset. (XLSX)
